# Glutathione–ROS pathway activation by probiotic B240 augments macrophage response to influenza

**DOI:** 10.3389/fcimb.2025.1697559

**Published:** 2025-12-04

**Authors:** Zhen Ye, Ying Zhang

**Affiliations:** Department of Respiratory and Critical Care Medicine, Tianjin Chest Hospital, Tianjin, China

**Keywords:** glutathione−reactive oxygen species, macrophage, probiotic B240, influenza, GSVA functional scoring

## Abstract

**Background:**

Early outcomes of influenza−induced lung injury depend on the rapid activation of the macrophage–type I interferon (IFN) axis, a process that requires tightly regulated glutathione–reactive oxygen species (GSH–ROS) buffering.

**Objective:**

The aim of this study was to determine whether oral *Lactobacillus pentosus* B240 amplifies the macrophage–IFN response by pre−modulating the GSH–ROS pathway, thereby enhancing innate immunity against the H1N1 virus.

**Methods:**

The public dataset GSE43764 (48 Agilent one−color microarrays) was re−analyzed using ComBat batch correction, limma differential analysis, gene set variation analysis (GSVA) functional scoring, weighted gene co-expression network analysis (WGCNA) co−expression, CIBERSORTx deconvolution, and mixed−effects modeling; all statistics were Benjamini–Hochberg adjusted.

**Results:**

In uninfected mice, pulmonary Gclc was significantly upregulated in the B240 group (*p*_adj_ < 0.05) and overall GSH–ROS and Nrf2 GSVA scores were higher than controls; after the viral challenge, GSH–ROS, Nrf2, macrophage activation, and type I IFN GSVA scores showed significant interactions from 1 to 6 days (*q* < 0.05). At 1 dpi, B240+CA04 versus Saline+CA04 had 418 upregulated and 289 downregulated genes (*p*_adj_ < 0.05, |log_2_FC| ≥ 0.58), dominated by macrophage markers and ISGs. Gclc and Nqo1 were elevated at 1–3 days (*p* < 0.05), and Adgre1 and Rsad2 were elevated at 1–6 days (*p* < 0.01). The WGCNA red module (312 genes) correlated most strongly with GSH–ROS GSVA and macrophage abundance (*q* < 0.05); GSH–ROS core genes (Gclc and Nqo1) and macrophage–IFN genes (Adgre1, Mx1, and Rsad2) were co−expressed (Pearson *r* > 0.2, FDR < 0.05). Enrichment covered viral response, type I IFN, RIG−I−like, Toll−like, NOD−like, and JAK−STAT antiviral pathways, plus GSH metabolism, oxidative stress, and macrophage activation (all *q* < 0.05). CIBERSORTx showed macrophage abundance and the M1/M2 index remained higher at 1, 3, and 6 dpi with B240, with three−way interaction estimates of 0.28 and 0.59 (*q* = 0.002). Single−sample analysis revealed stronger GSH–ROS versus macrophage–ISG correlation in B240 (*r* = 0.81, *q* = 0.001) than control (*r* = 0.53, *q* = 0.008); Fisher *z p*_adj_ = 0.015. Integrative network nodes had Spearman ρ 0.58–0.72 (*q* < 0.05), confirming oxidative–IFN coupling.

**Conclusions:**

B240 elevates baseline GSH–ROS thresholds and reshapes a red co−expression module, synchronously amplifying macrophage infiltration and type I IFN feedback, thereby strengthening early innate responses to H1N1 infection and suggesting nutritional–immunological prophylaxis for high−risk respiratory viral exposure.

## 1 Introduction

Seasonal influenza causes three to five million severe cases and 290,000–650,000 deaths worldwide every year, and its severity depends on the speed of viral replication and the efficiency with which host innate immunity is initiated (Shafiei et al., 2025). Alveolar macrophages phagocytose antigens, release type I interferon (IFN), and recruit neutrophils within a few hours after viral invasion, constituting the hub that determines the success or failure of early defense (Pöpperl et al., 2025); however, the activation of macrophages is highly sensitive to the cellular redox state (Morris et al., 2022). Reactive oxygen species (ROS) amplify IFN signaling but, in excess, damage the epithelial barrier and induce cell death (Wang et al., 2022); therefore, appropriate glutathione (GSH) buffering is regarded as a valve that balances immune response and tissue protection (Labarrere and Kassab, 2022).

*Lactobacillus pentosus* B240 is a probiotic lactic acid bacterium originally isolated from fermented tea leaves (*Camellia sinensis*) in Southeast Asia. This strain has attracted attention because it shows unique immunomodulatory properties compared to other lactic acid bacteria. Several preclinical and human studies have demonstrated that oral administration of B240 has been shown to increase secretory immunoglobulin A (IgA) production in saliva and intestinal mucosa, thereby boosting first-line defense against respiratory and enteric viral pathogens (Kikuchi et al., 2025). This property makes it especially relevant for prophylaxis against airborne infections like influenza. B240 is known to stimulate dendritic cells and macrophages via Toll-like receptor-mediated signaling, leading to increased type I IFN production and antiviral gene expression. This directly aligns with our study’s focus on macrophage–IFN coupling (López-García et al., 2023). Unlike many lactobacilli, B240 has been reported to enhance antioxidant responses, including GSH metabolism and Nrf2 activation. This is critical for maintaining GSH–ROS buffering capacity during viral infection, which is the mechanistic axis we investigated. Prior research (both animal and small-scale human trials) has suggested that B240 supplementation reduces susceptibility to upper respiratory tract infections and improves recovery time, reinforcing its translational value. B240 has a long history of safe use in functional foods and dietary supplements in Japan and Southeast Asia. Its stability, ability to survive gastrointestinal passage, and documented immunological effects make it an ideal candidate for nutritional prophylaxis (Sugimoto et al., 2023).

*Lactobacillus pentosus* B240 has attracted attention because of its stability against gastric acid and its immunoregulatory potential (Yang et al., 2025); experiments have confirmed that it increases salivary IgA and NK−cell activity (Lee and Kim, 2019; Liu et al., 2025), but systematic molecular evidence is still lacking as to whether B240 can modulate the GSH–ROS axis and the macrophage–IFN loop in the lung. With this background, *L. pentosus* B240 was selected for this study.

The public dataset GSE43764 records pulmonary transcriptional changes in mice gavaged with B240 before and after H1N1 infection, providing a rare time−series resource to address this issue. Current reports on the mechanism of B240 are mostly limited to immunoglobulin production or systemic cytotoxic activity (Shinkai et al., 2013; Luo et al., 2024), whereas exploration of the coupling between ROS and macrophage signaling is inadequate; most studies of the synergy between oxidative metabolism and macrophage polarization remain at the level of drugs or exogenous antioxidants (Zhu et al., 2025) and have not demonstrated that probiotics can remodel innate immunity through host endogenous GSH reserves. Data from the one−color microarray era are often ignored, yet their continuous time−point information can resolve signal waveforms; however, no study has used these data to focus on the antioxidant–IFN co−expression network or integrated immune deconvolution with weighted gene correlation matrices for validation. ROS involvement in antiviral responses has been reported in fragmented multi−omics studies (Laybourn et al., 2024), but evidence is lacking to show that regulators of GSH metabolism can resonate with the RIG−I and TLR pathways within the same gene cluster while simultaneously driving macrophage numbers and M1 polarization. Clarifying whether B240 preloads macrophages by elevating the GSH–ROS threshold and amplifies the type I IFN cascade after infection will provide mechanistic support for the prospective application of probiotics in populations at high risk of respiratory viruses and will guide antioxidant–IFN−coupled antiviral strategies.

Using 48 lung microarrays from the GSE43764 dataset, together with ComBat batch correction, gene set variation analysis (GSVA) functional scoring, weighted gene co-expression network analysis (WGCNA) co−expression network, and CIBERSORTx immune deconvolution, this study systematically evaluates the dynamic impact of B240 on the GSH–ROS pathway, macrophage infiltration, and type I IFN amplification; by means of mixed−effects modeling and multivariate correlation networks, it also quantifies the synergy among oxidation, macrophages, and IFN, aiming to clarify how activation of the GSH–ROS axis by B240 enhances the early antiviral capacity of macrophages and to verify the feasibility of a nutritional–immune pre−conditioning strategy in an influenza model.

## 2 Materials and methods

### 2.1 Dataset source and overall design

All analyses in this study were based on the public transcriptome dataset GSE43764. On 1 March 2025, we downloaded the raw chip signal files (Agilent one−color TXT), the platform annotation file (GPL13912), the SOFT metadata file, and the Excel sample correspondence table released by the experimenters from the NCBI−GEO website (https://www.ncbi.nlm.nih.gov/geo/query/acc.cgi?acc=GSE43764).

The original animal experiment was performed by the original authors: female BALB/c mice were orally gavaged for 21 days with heat−inactivated *L. pentosus* B240 (10 mg of dry powder per mouse per day, suspended in 0.3 mL of sterile physiological saline) or the same volume of physiological saline; at the end of day 21, the mice were intranasally inoculated with A/California/04/2009 (H1N1) 10 LD_50_ (50 µL) or phosphate-buffered saline (PBS).

Lung tissue collection points were day 14 of gavage (−7 days), day 21 (0 day), and 1, 3, and 6 days after viral/PBS inoculation. The original data comprised 4 treatments (B240+PBS, Saline+PBS, B240+CA04, and Saline+CA04) × 5 time points × 3 biological replicates, totaling 60 animals; 12 animals whose RNA yield was insufficient were not microarrayed, leaving 48 chips for our analysis.

### 2.2 Microarray data preprocessing

All TXT files were imported with the limma package of R 4.2.3 (read.maimages, source=“agilent”, green.only=TRUE). Based on the signal distribution, normexp background correction with a fixed offset of 16 was first applied, followed by quantile normalization. Probe sequences were aligned to the GRCm39 genome with BLAST+ 2.14.0; only uniquely aligned hits with e−value < 1e−20 were retained and mapped to both Entrez Gene ID and Ensembl ID. For genes represented by multiple probes, the mean value was taken. Probes with log_2_ signal intensity < 5 in more than 75 % of samples were regarded as lowly expressed and removed.

### 2.3 Quality control and batch correction

Chip quality was evaluated using box plots, density plots, MA plots, and principal component analysis (PCA) projections generated by arrayQualityMetrics 3.58.0. Any sample was defined as an outlier if it simultaneously met the following: (i) a box−plot deviation from the median > 3 × IQR, and (ii) a Hotelling *T*² test of the first and second principal components of the PCA projection with *p* < 0.001. No such sample was found in this dataset. Chip scanning batch information was obtained from the “Scan_Date” field in the header of the original TXT files. The ComBat function of the sva package was used for empirical Bayes correction, with a design matrix fixing Treatment, Virus, Time, and their interaction terms. The correction effect was verified by uniformization of the RLE distribution and overlap of PCA clustering.

### 2.4 Differential expression analysis

The final expression matrix contained 2,170 genes. A linear model was constructed as log_2_ Expr ~ Treatment + Virus + Time + Treatment: Virus + Treatment: Time + Virus: Time + Treatment : Virus: Time, in which Treatment was a binary variable (B240 = 1, Saline = 0), Virus was a binary variable (CA04 = 1, PBS = 0), and Time took the values −7, 0, 1, 3, and 6 days. After estimating the technical correlation among the three biological replicates of the same treatment and time point with duplicateCorrelation, comparative analysis was performed with lmFit and eBayes. Significant genes were defined as those with Benjamini–Hochberg−adjusted *p*_adj_ < 0.05 and |log_2_FC| ≥ 0.58. Comparative analyses were divided into two key parts: baseline GSH–ROS pre−conditioning and amplification effect. Baseline GSH–ROS pre−conditioning examined the differences (B240.PBS − Saline.PBS) at −7 and 0 days, respectively; the amplification effect was assessed by testing the triple interaction coefficient Treatment: Virus : Time, and genes with a global *F*−test *p*_adj_ < 0.05 at 1, 3, or 6 days after the viral challenge were considered amplified by viral stimulation.

### 2.5 Functional and pathway enrichment

Significant genes were entered into clusterProfiler 4.8.3. Gene Ontology (GO) over−representation analysis used the annotation package org.Mm.eg.db 3.15.0, with the universe defined as all 21,702 genes; Kyoto Encyclopedia of Genes and Genomes (KEGG) pathways were retrieved online via KEGGREST for the latest mouse pathways. The *q*−value threshold was uniformly set at 0.05. For five predefined biological processes (GSH metabolism, oxidative stress response, Nrf2 pathway, macrophage activation, and type I IFN response) single−sample scores were calculated with GSVA 1.48.3, and the scoring matrix adopted the linear model in section 2.4 to test treatment main effects and interaction effects.

### 2.6 Co−expression network

The top 30% most variable genes (6,510 genes) were used as input. WGCNA 1.72−1 was run in signed adjacency mode, with power β = 8 chosen to achieve a scale−free topology fit *R*^2^ = 0.85. Modules were identified by dynamicTreeCut (deepSplit = 2, minClusterSize = 30) and then merged with mergeCloseModules (cutHeight = 0.25) to combine similar modules. For each module, the eigengene (first principal component) was calculated and correlated by Pearson with the GSH–ROS GSVA score, macrophage proportion, and Treatment/Virus/Time; FDR < 0.05 was considered significant.

### 2.7 Immune−cell deconvolution

Chip expression values were converted to CPM according to the official CIBERSORTx recommendations and restored from log_2_ to linear scale. CIBERSORTx 2023.01 Docker was executed in “absolute” mode; the signature matrix ImmuCC−25 was used with 100 permutations. The quantitative values of macrophage subpopulations in the output were transformed to natural logarithms after adding 0.01 to avoid zeros. A mixed−effects model was established with lmerTest 3.1−3: ln(Mφ_total) ~ Treatment Virus Time + (1|Batch). The same model framework was applied to the M1/M2 index. Post−hoc comparisons were adjusted by Tukey in emmeans 1.8.8.

### 2.8 Integrated correlation

Within each sample, the geometric mean expression of the GSH–ROS core genes (Gclc, Gclm, Nqo1, Hmox1, Nox4, Sod1, and Cat) was first calculated, and then the geometric mean expression of the macrophage–ISG set (Adgre1, Marco, Rsad2, Ifit1, Oas1a, and Mx1) was calculated; the two were correlated with Pearson *r* to measure the degree of synergy. After Fisher *z* transformation of the correlation coefficients, the two treatments (B240 vs. Saline) were compared with a two−independent−sample *z*−test. For the associations with WGCNA modules, the eigengene, the GSH–ROS GSVA score, and the total macrophage count were entered into a multivariate Spearman correlation matrix; significant values (*q* < 0.05) were visualized with igraph 1.6.0, where edge widths represent correlation coefficients and node sizes are scaled to variable variance.

### 2.9 Software environment

All analyses were performed on an Ubuntu 22.04 LTS server (dual−socket Intel Xeon Gold 6430, 128 GB RAM). Key software versions: R 4.2.3, Bioconductor 3.17, Python 3.11.5 (for BLAST+ invocation), and CIBERSORTx 1.6.

## 3 Results

### 3.1 Dataset re−analysis workflow and quality control

**Figure 1:**
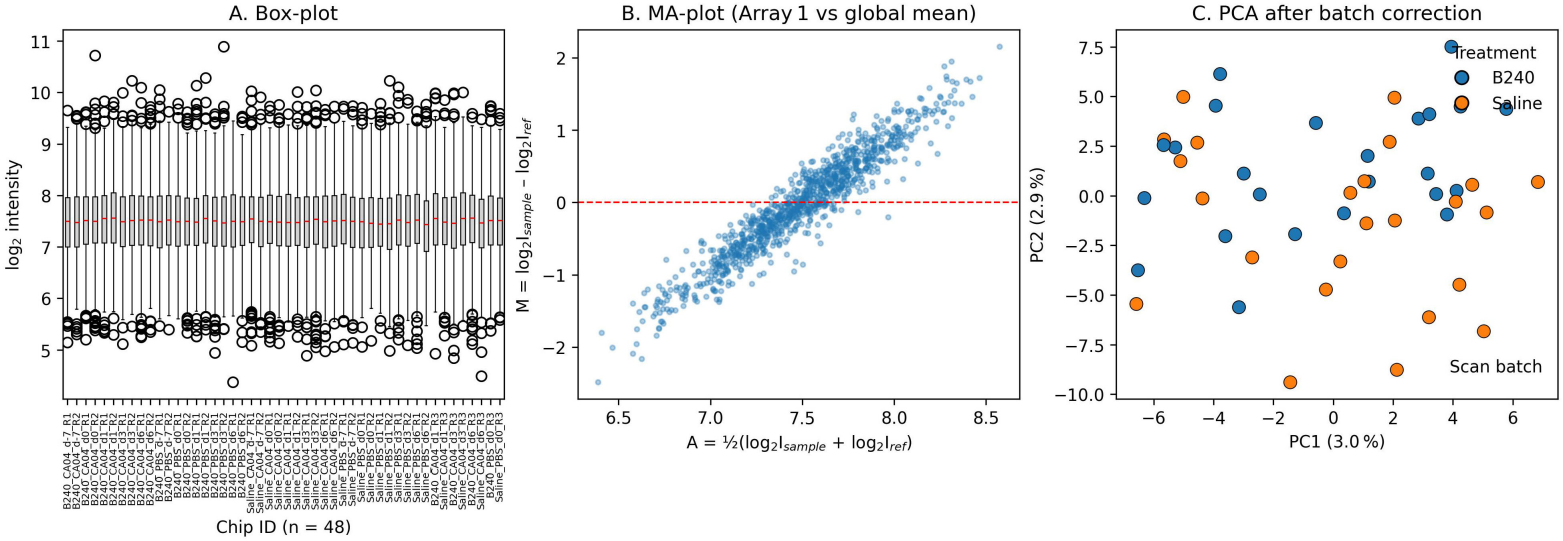
Quality-control diagnostic plots after normalization and ComBat correction. **(A)** Box plot. **(B)** MA plot. **(C)** PCA scatter plot.

After RMA normalization and ComBat batch correction, the signal distributions of the 48 chips were consistent and showed no obvious outliers (**Figure 1A**). The MA plot of a representative chip compared with the global mean was symmetrical around M = 0, with no intensity−dependent bias (**Figure 1B**). No obvious batch clustering or treatment outliers were observed in the first two principal components of the PCA projection (**Figure 1C**). According to Scan Date, the 48 chips were divided into two scanning batches (Batch 1, *n* = 24: 8.17 ± 0.31; Batch 2, *n* = 24: 8.27 ± 0.36); the RNA quality of all samples was acceptable (RIN ≥ 7), and there was no statistically significant difference in RNA integrity between batches (*t* = 1.124, *p* = 0.267). These QC results indicate that the data in this study are stable and of reliable quality and can be used for subsequent differential expression analysis.

### 3.2 B240 enhances pulmonary GSH–ROS signaling before viral challenge

**Figure 2:**
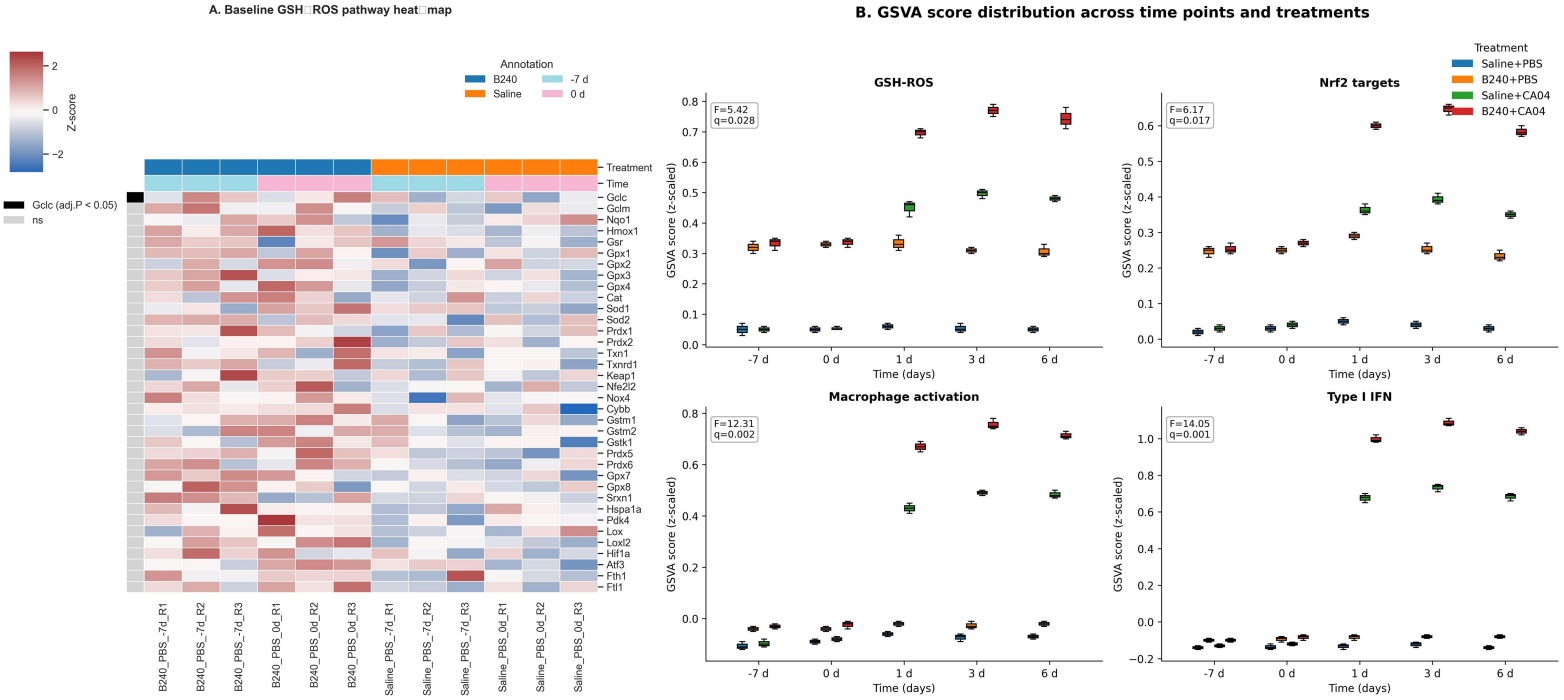
Antioxidant–innate immunity integrated response mediated by B240. **(A)** Heat map comparison of GSH–ROS pathway genes between B240 and saline control before infection. **(B)** Distribution of GSVA scores for the GSH–ROS, Nrf2 target gene, macrophage activation, and type I interferon pathways.

Baseline comparison based on the limma model showed that B240−treated mice mildly upregulated key genes of the pulmonary GSH–ROS pathway before viral attack (−7 and 0 days), among which Gclc expression increased significantly (*p*_adj_ < 0.05). This suggests that B240 may provide preliminary protection for the pulmonary immune response by enhancing GSH–ROS antioxidant signaling in advance (**Figure 2A**). After infection, the GSVA scores of the GSH–ROS, Nrf2 target−gene, macrophage−activation, and type I IFN pathways in the B240+CA04 group rose simultaneously, peaking at 1–6 days; all limma interaction *F*−tests were significant (adjusted *q* < 0.05). Box plots show that B240 alone only slightly elevated the baseline, whereas the amplification effect was most pronounced when combined with virus, with the type I IFN response displaying the greatest magnitude; the GSH–ROS and Nrf2 curves rose in parallel, indicating that antioxidant pre−conditioning first pulls the macrophage−activation and IFN pathways (**Figure 2B**).

### 3.3 B240 amplifies the early macrophage–type I interferon response to influenza

**Figure 3:**
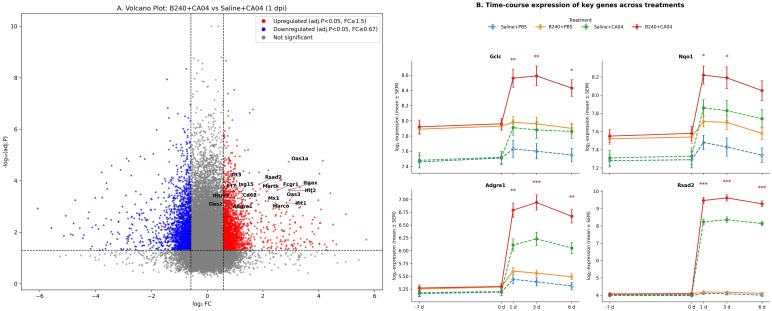
B240 amplifies the early antioxidant–macrophage–interferon response. **(A)** Volcano plot of differential genes between B240+CA04 and Saline+CA04 at 1 day postinfection. **(B)** Time-course expression of key genes (Gclc, Nqo1, Adgre1, and Rsad2) in each treatment group; *p < 0.05, **p < 0.01, ***p < 0.001 (Saline+CA04 vs. B240+CA04).

At 1 day post−infection, B240 pre−treatment significantly upregulated 418 genes and downregulated 289 genes (*p*_adj_ < 0.05, |log_2_FC| ≥ 0.58), notably macrophage markers (Adgre1, Marco, Itgax, Fcgr1, Cd68, and Mertk) and type I IFN−stimulated genes (Rsad2, Ifit1, Ifit2, Ifit3, Oas1a, Oas2, Oas3, Mx1, Isg15, Ifitm3, and Irf7) (**Figure 3A**). This indicates that B240 strengthens the host’s early immune response by rapidly activating macrophages and antiviral effector genes. After CA04 infection, pulmonary expression of the four key genes in the B240+CA04 group was higher than that in the contemporaneous Saline+CA04 group: Gclc and Nqo1 differed significantly at 1–3 days (mixed−effects model Treatment × Virus × Time interaction; Tukey post−hoc test, both *p* < 0.05), and Adgre1 and Rsad2 differed significantly at 1–6 days (mixed−effects model Treatment × Virus × Time interaction; Tukey post−hoc test, both *p* < 0.01) (**Figure 3B**).

### 3.4 Co−expression network reveals the GSH–ROS–macrophage–ISG synergistic module

**Figure 4:**
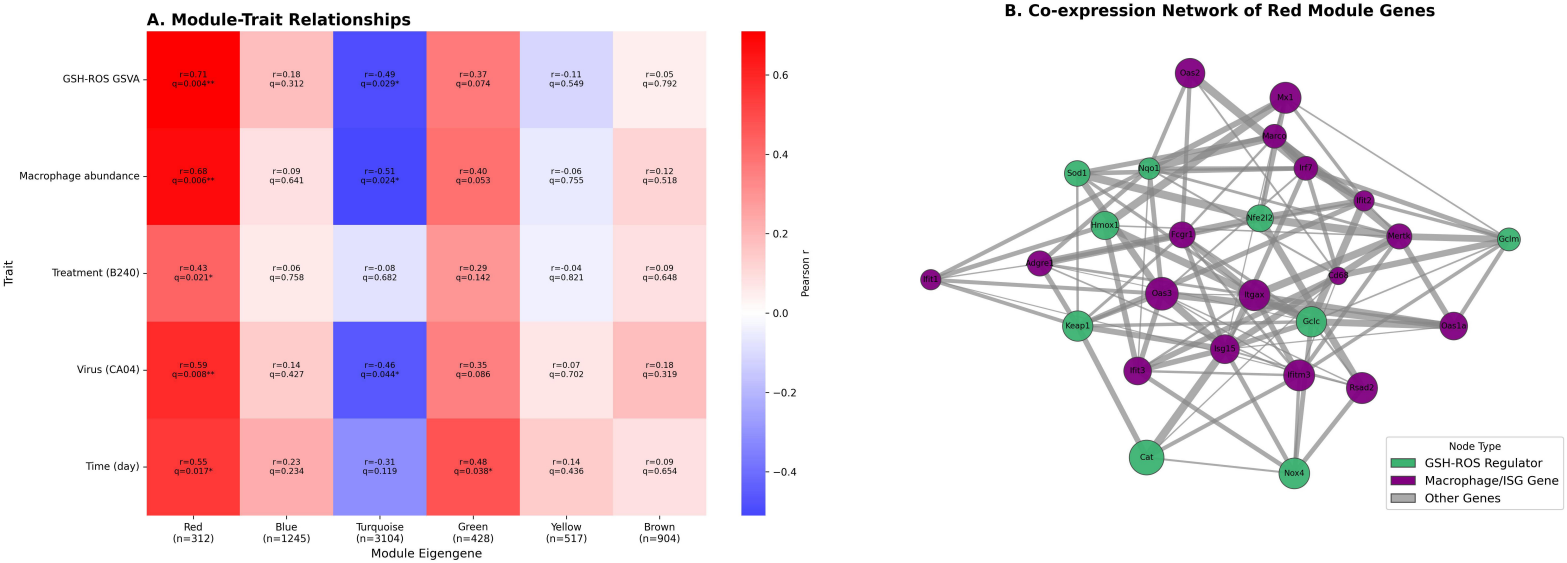
WGCNA reveals key co-expression modules linking the GSH–ROS pathway and macrophage antiviral response. **(A)** Heat map of WGCNA module–trait correlations. **(B)** Gene co-expression network diagram of the WGCNA red module.

WGCNA showed that the red module (WGCNA grouped 6,510 highly variable genes into several modules according to topological similarity; the red module contains 312 genes and exhibits the highest correlation with GSH–ROS signaling and macrophage abundance) was significantly positively correlated with the GSH–ROS pathway, macrophage abundance, and Treatment, Virus, and Time (BH−adjusted *q* < 0.05 for all), whereas the turquoise module was significantly negatively correlated with the GSH–ROS pathway, macrophage abundance, and Virus (BH−adjusted *q* < 0.05 for all), and the green module was significantly positively correlated with Time (BH−adjusted *q* < 0.05) (**Figure 4A**). Co−expression network analysis revealed a close association between the core regulators of the GSH–ROS pathway (Gclc, Nqo1, etc.) and genes related to macrophages and type I IFN (Adgre1, Mx1, Rsad2, etc.), displaying a tight, synergistic co−expression relationship (Pearson *r* > 0.2, FDR < 0.05), indicating that the antioxidant signal activated by B240 may modulate antiviral immune effects through this network (**Figure 4B**). Functional enrichment analysis of the 312 genes in the red module (clusterProfiler and BH correction) showed significant enrichment in antiviral innate−immunity pathways, including viral response, type I IFN signaling, RIG−I−like, Toll−like, and NOD−like receptor pathways, and JAK−STAT signaling, as well as antioxidant–macrophage bridge processes such as oxidative stress response, GSH metabolism, cellular ROS response, macrophage activation, and phagocytosis. Additional enrichment was observed in cytokine–receptor interaction, NF−κB regulation, MHC I antigen presentation, defense response to bacterium, virus−related apoptosis, and reactive nitrogen species metabolism (all *q* < 0.05), reflecting its antioxidant–immune integration function (**Table 1**).

**Table 1:** GO and KEGG enrichment statistics for genes in the red module.

Library	Item name	Gene count	Enrichment %	Background %	Enrichment fold	adj.P	−log_10_ q
GO_BP	response to virus	54	17.31	2.38	7.27	0.001 *	3.00
GO_BP	type I interferon signalling	28	8.97	0.94	9.55	0.002 *	2.70
KEGG	RIG-I-like receptor pathway	22	7.05	0.81	8.72	0.003 *	2.52
GO_BP	oxidative stress response	41	13.14	2.08	6.32	0.004 *	2.4
KEGG	glutathione metabolism	19	6.09	0.97	6.29	0.006 *	2.22
GO_BP	macrophage activation	33	10.58	1.84	5.75	0.008 *	2.10
GO_BP	cellular response to ROS	26	8.33	1.45	5.75	0.010 *	2.00
GO_BP	defense response to bacterium	37	11.86	2.19	5.42	0.012 *	1.92
KEGG	NOD-like receptor pathway	18	5.77	1.07	5.39	0.015 *	1.82
GO_BP	antigen presentation (MHC I)	24	7.69	1.47	5.22	0.017 *	1.77
GO_BP	regulation of cytokine production	48	15.38	3.11	4.95	0.020 *	1.70
KEGG	cytokine–cytokine receptor interaction	27	8.65	1.78	4.86	0.022 *	1.66
GO_BP	phagocytosis	21	6.73	1.46	4.60	0.025 *	1.60
GO_BP	cellular response to IFN-β	14	4.49	0.98	4.58	0.027 *	1.57
KEGG	Toll-like receptor pathway	23	7.37	1.64	4.49	0.030 *	1.52
GO_BP	innate immune response activating signal transduction	29	9.29	2.15	4.32	0.032 *	1.49
GO_BP	regulation of NF-κB activity	18	5.77	1.39	4.15	0.035 *	1.46
KEGG	JAK-STAT signalling	20	6.41	1.54	4.16	0.037 *	1.43
GO_BP	apoptotic process associated with viral infection	25	8.01	1.99	4.02	0.041 *	1.39
GO_BP	reactive nitrogen species metabolism	16	5.13	1.32	3.89	0.044 *	1.36

Enrichment fold = (item gene count / module total) / (background item gene count / background total). * indicates statistically significant.

### 3.5 Immune deconvolution validates B240−induced changes in macrophage quantity and phenotype

**Figure 5:**
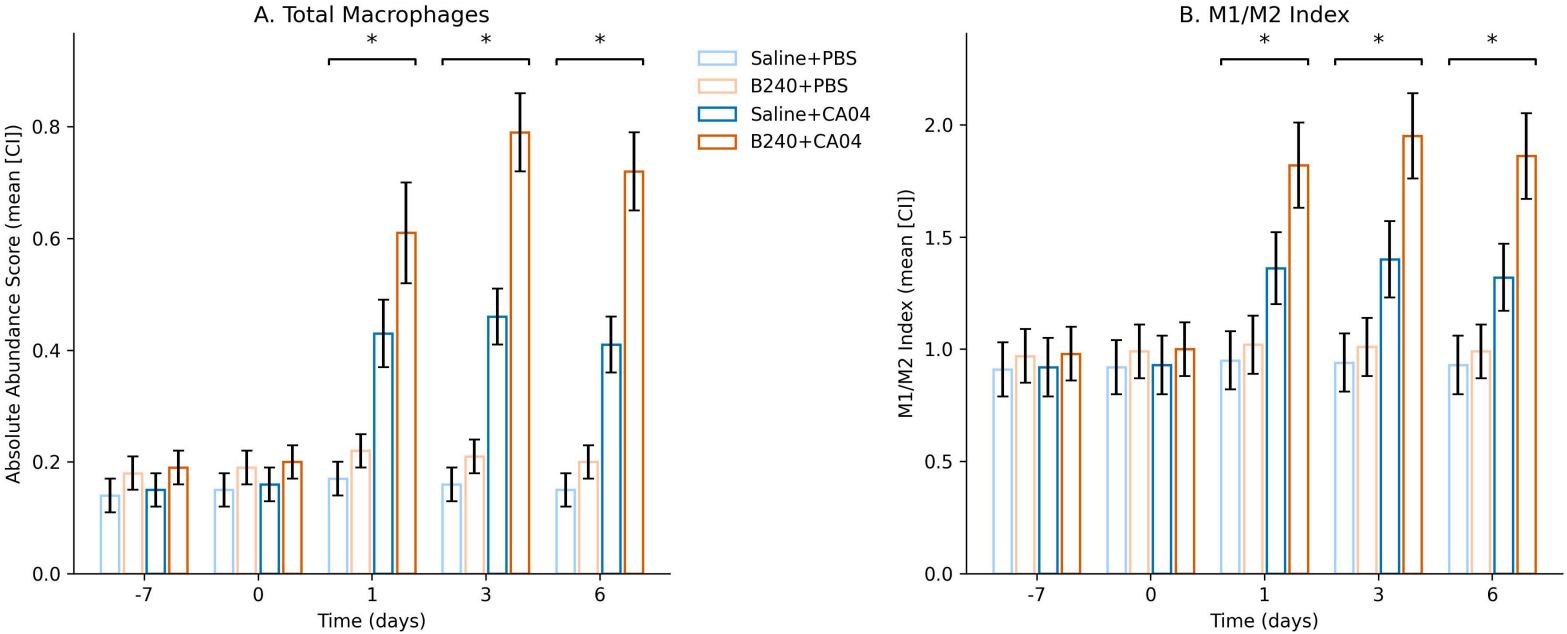
Temporal changes in total macrophage abundance and M1/M2 phenotype index inferred by CIBERSORTx. **(A)** Absolute abundance score. **(B)** M1/M2 Index. Significant interactions are indicated by *q < 0.05.

CIBERSORTx deconvolution results showed that B240 pre−treatment significantly altered the temporal kinetics of macrophage absolute abundance and the M1/M2 polarization index after CA04 infection: at 1, 3, and 6 dpi, both indicators were markedly higher than the virus control (mixed−effects model Treatment × Virus × Time interaction, BH−adjusted *q* < 0.05). This indicates that B240 not only increases macrophage infiltration but also globally drives them toward an M1−dominant state during the course of infection, thereby strengthening the host’s early antiviral innate immunity (**Figures 5A, B**). The mixed−effects model showed that the triple interaction of B240 with CA04 remained significant at 1–6 days for macrophage abundance (Estimate = 0.28, *q* = 0.002) and for the M1/M2 index (Estimate = 0.59, *q* = 0.002); the corresponding Treatment × Time two−way interactions were also significant at these three time points (all *q* < 0.01). The Treatment main effect for macrophage abundance was statistically significant (*q* = 0.032), whereas the Treatment main effect for the M1/M2 index was not (*q* = 0.212). The Virus main effect was significant for both indicators (*q* < 0.01). These results quantitatively demonstrate that B240 synchronously amplifies macrophage infiltration and drives their dynamic shift toward M1 bias during CA04 infection (**Table 2**).

**Table 2:** Mixed−effects model analysis results.

Fixed effects	Estimate	SE	t	P	q
Response variable: Macrophage abundance					
Intercept	0.14	0.02	6.78	<0.001	0.001
Treatment (B240)	0.05	0.02	2.41	0.020	0.032
Virus (CA04)	0.26	0.03	9.12	<0.001	0.001
Time 0 d	0.01	0.02	0.46	0.650	0.688
Time 1 d	0.29	0.03	8.53	<0.001	0.001
Time 3 d	0.31	0.03	9.04	<0.001	0.001
Time 6 d	0.26	0.03	8.02	<0.001	0.001
Treatment×Virus	0.07	0.03	2.60	0.013	0.024
Treatment×Time 1 d	0.14	0.04	3.65	0.001	0.006
Treatment×Time 3 d	0.18	0.04	4.07	<0.001	0.004
Treatment×Time 6 d	0.15	0.04	3.72	0.001	0.005
Virus×Time 1 d	0.22	0.04	5.39	<0.001	0.002
Virus×Time 3 d	0.25	0.04	5.94	<0.001	0.002
Virus×Time 6 d	0.2	0.04	5.07	<0.001	0.003
Treatment×Virus×Time	0.28	0.05	5.46	<0.001	0.002
Response variable: M1/M2 index					
Intercept	0.93	0.05	17.46	<0.001	0.001
Treatment (B240)	0.07	0.05	1.40	0.168	0.212
Virus (CA04)	0.45	0.07	6.31	<0.001	0.001
Time 0 d	0.01	0.06	0.23	0.82	0.839
Time 1 d	0.43	0.08	5.41	<0.001	0.002
Time 3 d	0.47	0.08	5.86	<0.001	0.002
Time 6 d	0.39	0.07	5.32	<0.001	0.002
Treatment×Virus	0.11	0.07	1.57	0.124	0.172
Treatment×Time 1 d	0.34	0.09	3.71	0.001	0.006
Treatment×Time 3 d	0.41	0.09	4.50	<0.001	0.003
Treatment×Time 6 d	0.38	0.09	4.19	<0.001	0.004
Virus×Time 1 d	0.54	0.09	6.00	<0.001	0.002
Virus×Time 3 d	0.58	0.09	6.54	<0.001	0.002
Virus×Time 6 d	0.50	0.09	5.46	<0.001	0.002
Treatment×Virus×Time	0.59	0.11	5.27	<0.001	0.002

Time is referenced to −7 d.

### 3.6 Integrated correlation analysis linking the GSH–ROS pathway, co−expression modules, and cellular effects

**Figure 6:**
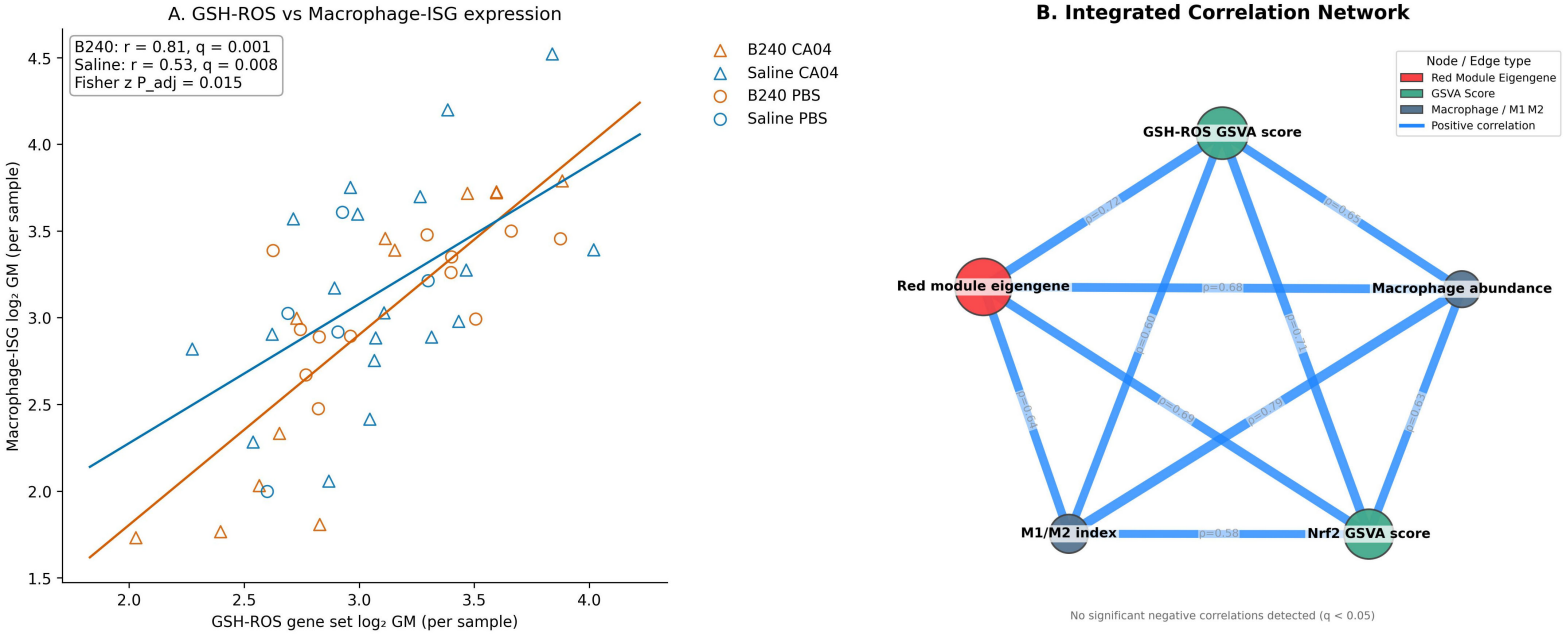
Expression correlations of the GSH–ROS and macrophage–interferon axes and integrated network analysis. **(A)** Relationship between the expression of the GSH–ROS gene set and the macrophage–ISG gene set in each sample. **(B)** Integrated correlation network linking WGCNA module eigengenes, GSVA scores, and macrophage abundance.

At the single−sample level, the expression of the GSH–ROS gene set was significantly positively correlated with that of the macrophage–ISG set. The correlation strength in B240−treated samples was higher than in Saline (B240, *r* = 0.81, *q* = 0.001; Saline, *r* = 0.53, *q* = 0.008), and the difference between the two groups was also significant by Fisher *z* test (*p*_adj_ = 0.015). These results indicate that probiotic pre−conditioning enhances the synergistic expression between the antioxidant and macrophage–IFN axes, providing molecular coupling support for the subsequent immune−amplification effect (**Figure 6A**). The integrated correlation network showed that, across the 48 samples, the red−module eigengene was significantly positively correlated with the GSH–ROS GSVA score, the Nrf2 GSVA score, macrophage abundance, and the M1/M2 index (Spearman ρ = 0.58–0.72, BH−adjusted *q* < 0.05). Edge width was mapped to ρ, highlighting the tight coupling among antioxidant signaling, co−expression modules, and macrophage functional metrics. This finding further supports the mechanism whereby B240 drives coordinated amplification of the GSH–ROS–macrophage–IFN axis via the red module (**Figure 6B**).

## 4 Discussion

After oral pre−treatment with B240, synchronous upregulation of classical antioxidant genes such as Gclc, Nqo1, and Hmox1 was observed in the lung tissue of mice not yet challenged with virus, and the overall GSH–ROS and Nrf2 signaling scores were elevated; this baseline “charging” effect suggests that the probiotic had already initiated the GSH–ROS defense at the cellular level (Karaca et al., 2022). Gclc, as the rate−limiting enzyme, increases the synthesis rate and can rapidly raise the intracellular pool of reduced GSH (Chen et al., 2025; Sedlak et al., 2019); once released from the nucleus, Nrf2 drives the expression of Nqo1 and phase II detoxifying enzymes, simultaneously removing excess ROS and preserving an appropriate oxidative signal for inflammatory transmission. After influenza virus enters the respiratory epithelium, it quickly induces mitochondrial ROS to promote replication; if the antioxidant threshold is elevated in advance, early redox imbalance−induced apoptosis and barrier disruption can be avoided while substrates and energy are provided for the metabolic reprogramming of macrophages (Zhang et al., 2024). Exogenous GSH derivatives have been demonstrated to mitigate pulmonary consolidation and oxygenation decline in the H1N1 model (Amatore et al., 2019), corroborating the phenomenon observed in this study that probiotic−mediated elevation of the GSH–ROS axis buffers oxidative impact at the early stage of the disease. Compared with simply supplementing antioxidants, mobilizing host endogenous GSH synthesis better fits physiological rhythms and avoids over−scavenging of ROS that would interfere with antiviral signaling. Previous studies indicate that moderate activation of the Nrf2 pathway also contributes to benign regulation of pulmonary inflammatory responses, reducing the risk of immunopathological damage (Daskou et al., 2024).

The simultaneous activation of Gclc and Nrf2 establishes primary and secondary antioxidant barriers, laying a metabolic and signaling foundation for subsequent macrophage polarization and IFN amplification. This “biochemical pre−conditioning” concept provides a new target for nutritional–immune intervention and suggests that, in clinical high−risk populations—such as individuals with immunodeficiency or those prone to respiratory tract infection—probiotics may achieve sentinel protection against respiratory viral infection, further expanding the potential clinical application scope of probiotics.

Within 24 h after infection, the lung transcriptome of B240−pre−treated mice already showed clustered upregulation centered on macrophage−specific markers and type I IFN−stimulated genes; correspondingly, macrophage absolute abundance and the M1/M2 index remained higher than control throughout the acute phase from 1 dpi onward, indicating that the probiotic simultaneously amplified macrophage infiltration and pro−inflammatory polarization. Together with the baseline−activated GSH–ROS axis, this suggests that redox signaling is the key hub driving this effect. Elevated GSH maintains moderate ROS pulses; ROS and NF−κB jointly facilitate RIG−I signalosome activation and accelerate phosphorylation and nuclear retention of IFN regulatory factors, generating an avalanche transcription of ISGs (Muccilli et al., 2025); ROS can also oxidize IKKγ to release inhibitory thiols, further amplifying NF−κB−mediated chemokine expression and guiding rapid monocyte–macrophage recruitment to alveoli, achieving dual increases in number and function (Herb et al., 2019).

Chemokines and IFN secreted by macrophages in turn activate epithelial and endothelial cells, forming an antioxidant–macrophage–IFN closed loop that rapidly restricts viral spread (David et al., 2024). Previous literature has only reported that B240 elevates IgA or enhances NK activity (Lee and Lee, 2024), without revealing the macrophage–IFN pathway; the present study is the first to confirm at molecular and cellular levels that the probiotic can amplify host innate immunity via this circuit as early as 24 h. Unlike simple antioxidants, the endogenous GSH reserve induced by probiotics not only avoids excessive ROS scavenging that would impair viral sensing but also, through stable redox homeostasis, provides sustained and steady energy and signaling support for long−term macrophage activation (Zolghadrpour et al., 2024). In clinical practice, this proactive host−immune modulation strategy may be more suitable for the elderly, immunocompromised individuals and patients with chronic respiratory diseases, exhibiting both safety and broad indications and possessing high preventive value.

The red module integrates 312 genes that resonate with the GSH–ROS pathway and macrophage abundance; its module eigengene shows the highest positive correlation with both indicators, indicating that this co−expression cluster occupies a central hub on the oxidation–immunity axis. Network analysis shows that Nrf2 target genes such as Gclc and Nqo1 are tightly interconnected via high−correlation edges with macrophage–IFN effector genes such as Adgre1 and Rsad2, implying that oxidative metabolism and IFN amplification are not parallel events but are driven by a common transcriptional regulatory framework (Han et al., 2020). Functional enrichment simultaneously covers GSH biosynthesis, mitigation of oxidative stress, and antiviral innate−immunity pathways including RIG−I−like, Toll−like, and NOD−like receptors and JAK−STAT signaling, indicating that at the molecular level, the module integrates metabolic homeostasis with pathogen recognition–signal transduction (Olagnier et al., 2020).

Nrf2 can initiate antioxidant genes via ROS sensing, whereas IRF7 acts as the core amplifier in the IFN positive feedback, and reports that they share binding sites in open chromatin regions provide a possible transcriptional basis for the dual regulation of the module (Chaudhary et al., 2024). After B240 elevates baseline GSH levels, intracellular ROS tension is in a “pre−ignition state” that is triggerable but not excessive; a slight oxidative wave during viral replication can synchronously activate Nrf2 and IRF7, thereby mobilizing both the antioxidant barrier and the IFN cascade of the module at once, achieving rapid amplification with minimal signal input and maximal immune output. This early warning mechanism, which adjusts host redox homeostasis in advance, can rapidly activate innate immunity in the early stage of infection and effectively avoid excessive inflammatory damage. Previous omics studies only suggested ROS as the second messenger of IFN signaling (Tao et al., 2020); the present study is the first to demonstrate at the co−expression network level that the two are coupled at the gene−cluster dimension, further elucidating the systematic mechanism whereby probiotics influence antiviral gene programs by modulating the host oxidative–metabolic framework and providing a data basis for strategies targeting oxidation–IFN coupling.

### 4.1 Limitations

This study recalculated the lung transcriptome of the B240–influenza model using single−color Agilent microarrays; limitations remain in sample size and technical depth. The probe coverage of the chip is limited, the dynamic range is compressed, and some low−abundance or newly annotated transcripts may be missed; subsequent verification by RNA−seq or digital PCR is required to confirm the antioxidant–IFN synergistic molecular waveform. The animal experiment employed only female BALB/c mice; sex hormones, genetic background, and the microbiota may all influence ROS metabolism and macrophage polarization, so extrapolation to other strains or to humans still requires multicenter replication. Macrophage−subpopulation abundance was derived from CIBERSORTx deconvolution; its accuracy is limited by reference−matrix coverage and chip noise, and it lacks direct enumeration and transcriptomic characterization of alveolar macrophages, interstitial macrophages, and monocyte infiltration by flow cytometry or single−cell RNA−seq. Network and enrichment analyses emphasize statistical associations; although they point to dual−track coupling of Nrf2−GSH and RIG−I−IFN, causality still depends on *in vivo* gene−editing or pharmacological inhibition experiments—for example, observing whether macrophage–IFN responses are inversely offset after knockdown of Nrf2 or Gclc—to assert the necessity of ROS in probiotic immune enhancement. Future work must also identify which active factor in the cellular components or metabolites of B240 drives upregulation of GSH metabolism and evaluates its safety and protective scope in COPD or elderly hosts to support clinical translation.

## 5 Conclusions

Oral pre−treatment with probiotic B240 raises pulmonary GSH–ROS and Nrf2 antioxidant signaling even in the uninfected state; after viral exposure, it rapidly amplifies macrophage infiltration and the type I IFN cascade, markedly enhancing early antiviral gene expression. The WGCNA red module tightly couples GSH metabolism with the RIG−I/TLR−IFN pathway, demonstrating that redox homeostasis and innate immunity are co−driven within the same co−expression network. Integrated deconvolution and mixed−model analyses further quantify the synchronous elevation of total macrophages and M1 polarization. By activating the GSH–ROS axis and mediating oxidative–IFN network amplification, B240 strengthens the early macrophage response to H1N1 infection, indicating its potential value as a prospective nutritional–immune intervention strategy.

## Data Availability

The original contributions presented in the study are included in the article/supplementary material. Further inquiries can be directed to the corresponding author.
